# Supplementary material for: Cutaneous anthrax outbreak associated with use of cattle hides and handling carcasses, Amudat District, Uganda, 2023–2024
Source: PLoS One. 2025 Nov 17;20(11):e0336769. doi: 10.1371/journal.pone.0336769 (PMC12622773; doi:10.1371/journal.pone.0336769)
Supplement: Attachment — Submitted filename: cutaneous anthrax.pdf [file pone.0336769.s002.pdf]

PLOS ONE

Cutaneous anthrax outbreak linked to sleeping on hides and slaughtering of suddenly deceased cattle, Amudat District, Uganda, December 2023–June 2024

--Manuscript Draft--

**Manuscript Number:**	PONE-D-25-41159
**Article Type:**	Research Article
**Full Title:**	Cutaneous anthrax outbreak linked to sleeping on hides and slaughtering of suddenly deceased cattle, Amudat District, Uganda, December 2023–June 2024
**Short Title:**	Cutaneous anthrax outbreak linked to hides and dead cattle in Amudat District, Uganda, 2023–2024
**Corresponding Author:**	Patrick Kwizera<br>NIPH: National Institute of Public Health<br>Kampala, UGANDA
**Keywords:**	Anthrax; Disease outbreaks; Cutaneous anthrax; Uganda
**Abstract:**	Background<br><br>On December 28, 2023, a 10-year-old in Amudat District presented with symptoms consistent with anthrax, marking the district's first suspected case. After several sporadic cases and laboratory confirmation on March 25, 2024, we investigated to assess the outbreak's magnitude, identify risk factors, and recommend prevention measures.<br><br>Methods<br><br>We defined a suspected cutaneous anthrax case as acute onset of ≥2 of the following: vesicles/eschar, skin itching, reddening, swelling, or regional lymphadenopathy, in Amudat residents from December 2023–June 2024. A confirmed case tested PCR-positive for Bacillus anthracis. We identified cases through household searches, medical record reviews, and snowballing. Human, animal, and environmental samples were tested. A case-control study involving 40 cases and 120 controls was conducted, using multivariable logistic regression to identify associated risk factors.<br><br>Results<br><br>We identified 102 suspected and 7 confirmed cases; none were fatal. The outbreak lasted 7 months, peaking in March 2024, with an overall attack rate of 167/100,000 (males: 201/100,000; females: 132/100,000). Sleeping on animal hides (OR=11; 95% CI: 2.6–47) and slaughtering suddenly deceased cattle (OR=5.3; 95% CI: 1.8–15) were significantly associated with anthrax. Risk increased with multiple exposures, with highest odds among those involved in slaughtering, skinning, and carrying meat (OR=19; 95% CI: 2.6–136). No livestock had been vaccinated prior to the outbreak.<br><br>Conclusion<br><br>The outbreak was linked to contact with animal hides and slaughtering of cattle that died suddenly. Delayed detection contributed to its spread. We recommended community education, livestock vaccination, and safe carcass handling to prevent future outbreaks.
**Order of Authors:**	Patrick Kwizera
	Richard Migisha
	Hannington Katumba
	Esther Nabatta
	Samuel Gidudu
	Benon Kwesiga

Powered by Editorial Manager® and ProduXion Manager® from Aries Systems Corporation

	Job Morukileng
	Alex Riolexus Ario
**Additional Information:**	
**Question**	**Response**
**Financial Disclosure**<br><br>Enter a financial disclosure statement that describes the sources of funding for the work included in this submission. Review the submission guidelines for detailed requirements. View published research articles from *PLOS ONE* for specific examples.<br><br>This statement is required for submission and **will appear in the published article** if the submission is accepted. Please make sure it is accurate.<br><br>**Funded studies**<br>Enter a statement with the following details:<br>• Initials of the authors who received each award<br>• Grant numbers awarded to each author<br>• The full name of each funder<br>• URL of each funder website<br>• Did the sponsors or funders play any role in the study design, data collection and analysis, decision to publish, or preparation of the manuscript?<br><br>Did you receive funding for this work?	The author(s) received no specific funding for this work.
**Competing Interests**<br><br>Use the instructions below to enter a competing interest statement for this submission. On behalf of all authors, disclose any competing interests that could be perceived to bias this work—acknowledging all financial support and any other relevant financial or non-financial competing interests.<br><br>This statement is required for submission and **will appear in the published article** if	The authors have declared that no competing interests exist.

Powered by Editorial Manager® and ProduXion Manager® from Aries Systems Corporation

the submission is accepted. Please make sure it is accurate and that any funding sources listed in your Funding Information later in the submission form are also declared in your Financial Disclosure statement.<br><br>View published research articles from *PLOS ONE* for specific examples.<br><br>**NO authors have competing interests**<br><br>Enter: *The authors have declared that no competing interests exist.*<br><br>**Authors with competing interests**<br><br>Enter competing interest details beginning with this statement:<br><br>*I have read the journal's policy and the authors of this manuscript have the following competing interests: [insert competing interests here]*<br><br>* typeset	
**Ethics Statement**<br><br>Enter an ethics statement for this submission. This statement is required if the study involved:<br><br>• Human participants<br>• Human specimens or tissue<br>• Vertebrate animals or cephalopods<br>• Vertebrate embryos or tissues<br>• Field research<br><br>Write "N/A" if the submission does not require an ethics statement.<br><br>General guidance is provided below. Consult the submission guidelines for detailed instructions. **Make sure that all information entered here is included in the Methods section of the manuscript.**	This outbreak investigation was in response to a public health emergency and was therefore determined to be non-research and therefore clinical trial number not applicable. The Ministry of Health (MoH) gave permission to investigate this outbreak. In agreement with the International Guidelines for Ethical Review of Epidemiological Studies by the Council for International Organizations of Medical Sciences (1991) and the Office of the Associate Director for Science, US CDC/Uganda, it was determined that this activity was not human subject research and that its primary intent was public health practice or disease control activity (specifically, epidemic or endemic disease control activity). This activity was reviewed by the US CDC and was conducted consistent with applicable federal law and CDC policy. §§See, e.g., 45 C.F.R. part 46, 21 C.F.R. part 56; 42 U.S.C. §241(d); 5 U.S.C. §552a; 44 U.S.C. §3501 et seq. All experimental protocols were approved by the US CDC human subjects review board (The National Institute for Occupational Safety and Health Institutional Review Board) and the Uganda Ministry of Health and were performed in accordance with the Declaration of Helsinki. Prior to data collection, informed consent was obtained from all the participants who were above 18 years (legal age in Uganda). For those below 18 years, consent was sought from their parents/guardians and assent was also obtained from them to participate in the study.

**Format for specific study types**<br><br>**Human Subject Research (involving human participants and/or tissue)**<br>• Give the name of the institutional review board or ethics committee that approved the study<br>• Include the approval number and/or a statement indicating approval of this research<br>• Indicate the form of consent obtained (written/oral) or the reason that consent was not obtained (e.g. the data were analyzed anonymously)<br><br>**Animal Research (involving vertebrate animals, embryos or tissues)**<br>• Provide the name of the Institutional Animal Care and Use Committee (IACUC) or other relevant ethics board that reviewed the study protocol, and indicate whether they approved this research or granted a formal waiver of ethical approval<br>• Include an approval number if one was obtained<br>• If the study involved *non-human primates*, add *additional details* about animal welfare and steps taken to ameliorate suffering<br>• If anesthesia, euthanasia, or any kind of animal sacrifice is part of the study, include briefly which substances and/or methods were applied<br><br>**Field Research**<br><br>Include the following details if this study involves the collection of plant, animal, or other materials from a natural setting:<br>• Field permit number<br>• Name of the institution or relevant body that granted permission	
**Data Availability**<br><br>Authors are required to make all data underlying the findings described fully available, without restriction, and from the time of publication. PLOS allows rare exceptions to address legal and ethical concerns. See the PLOS Data Policy and FAQ for detailed information.	No - some restrictions will apply

A Data Availability Statement describing where the data can be found is required at submission. Your answers to this question constitute the Data Availability Statement and **will be published in the article**, if accepted.<br><br>**Important:** Stating 'data available on request from the author' is not sufficient. If your data are only available upon request, select 'No' for the first question and explain your exceptional situation in the text box.<br><br>Do the authors confirm that all data underlying the findings described in their manuscript are fully available without restriction?	
**Describe where the data may be found in full sentences. If you are copying our sample text, replace any instances of XXX with the appropriate details.**<br><br>• If the data are **held or will be held in a public repository**, include URLs, accession numbers or DOIs. If this information will only be available after acceptance, indicate this by ticking the box below. For example: *All XXX files are available from the XXX database (accession number(s) XXX, XXX.*).<br>• If the data are all contained **within the manuscript and/or Supporting Information files**, enter the following: *All relevant data are within the manuscript and its Supporting Information files.*<br>• If neither of these applies but you are able to provide **details of access elsewhere**, with or without limitations, please do so. For example:<br><br>*Data cannot be shared publicly because of [XXX]. Data are available from the XXX Institutional Data Access / Ethics Committee (contact via XXX) for researchers who meet the criteria for access to confidential data.*<br><br>*The data underlying the results presented in the study are available from (include the name of the third party*	The datasets upon which our findings are based belong to the Uganda Public Health Fellowship Program. For confidentiality reasons, the datasets are not publicly available. The datasets can be availed upon reasonable request from the corresponding author with permission from the Uganda Public Health Fellowship Program. Request can be directed at: pkwizera@uniph.go.ug

and contact information or URL).<br>• This text is appropriate if the data are owned by a third party and authors do not have permission to share the data.<br><br>* typeset	
Additional data availability information:	

Powered by Editorial Manager® and ProduXion Manager® from Aries Systems Corporation

Click here to access/download;Manuscript;Ms_Anthrax Outbreak in Amudat_Kwizera.docx

Cutaneous anthrax outbreak linked to sleeping on hides and slaughtering of suddenly deceased cattle, Amudat District, Uganda, December 2023–June 2024

Authors: Patrick Kwizera[1*], Richard Migisha[1], Hannington Katumba[1], Esther Nabatta[1], Samuel Gidudu[1], Benon Kwesiga[1], Job Morukileng[1], Alex Riolexus Ario[1]

Institutional affiliations:

[1]Uganda Public Health Fellowship Program, Uganda National Institute of Public Health, Kampala, Uganda

Correspondence*: Email: pkwizera@uniph.go.ug

Target Journal: PLOS ONE

Abstract word limit: 300

Abstract word count: 249

Body word limit:

Body word count:

Tables and figures limit:

Tables and figures count: 7

Abstract

Background: On December 28, 2023, a 10-year-old in Amudat District presented with symptoms consistent with anthrax, marking the district's first suspected case. After several sporadic cases and laboratory confirmation on March 25, 2024, we investigated to assess the outbreak's magnitude, identify risk factors, and recommend prevention measures.

Methods: We defined a suspected cutaneous anthrax case as acute onset of ≥2 of the following: vesicles/eschar, skin itching, reddening, swelling, or regional

lymphadenopathy, in Amudat residents from December 2023–June 2024. A confirmed case tested PCR-positive for *Bacillus anthracis*. We identified cases through household searches, medical record reviews, and snowballing. Human, animal, and environmental samples were tested. A case-control study involving 40 cases and 120 controls was conducted, using multivariable logistic regression to identify associated risk factors.

Results: We identified 102 suspected and 7 confirmed cases; none were fatal. The outbreak lasted 7 months, peaking in March 2024, with an overall attack rate of 167/100,000 (males: 201/100,000; females: 132/100,000). Sleeping on animal hides (OR=11; 95% CI: 2.6–47) and slaughtering suddenly deceased cattle (OR=5.3; 95% CI: 1.8–15) were significantly associated with anthrax. Risk increased with multiple exposures, with highest odds among those involved in slaughtering, skinning, and carrying meat (OR=19; 95% CI: 2.6–136). No livestock had been vaccinated prior to the outbreak.

Conclusion: The outbreak was linked to contact with animal hides and slaughtering of cattle that died suddenly. Delayed detection contributed to its spread. We recommended community education, livestock vaccination, and safe carcass handling to prevent future outbreaks.

Keywords: Anthrax, Disease outbreaks, Cutaneous anthrax, Uganda

Background

Anthrax is a zoonotic disease caused by *Bacillus anthracis*, a gram-positive, aerobic, spore-forming bacterium that affects both animals and humans (1). Livestock typically become infected through ingestion of contaminated feed or grazing on land harbouring dormant spores, which then germinate into active bacteria within the host Humans

primarily acquire the infection through contact with infected animals or their products, such as meat, hides, skin, and bones. Anthrax manifests in three primary forms in humans: cutaneous, gastrointestinal and inhalational, each associated with a distinct route of exposure and varying incubation periods: cutaneous (2–7 days), inhalational (1–6 days), and gastrointestinal (1–6 days) (2). Cutaneous anthrax is the most frequently reported form, accounting for approximately 95% of human cases and may lead to fatality rates as high as 20% without treatment (3,4). Globally, anthrax continues to pose a significant public health threat, especially in underserved rural regions across Africa and Asia, where an estimated 20,000 to 100,000 cases occur annually(4,5). The World Health Organization (WHO) recognizes anthrax as one of the neglected zoonotic diseases due to its persistent burden on health and livelihoods in marginalized communities(4).

In Uganda, close human-animal interactions, particularly in pastoralist regions, increase the risk of zoonotic disease transmission(6). Between January 2017 and April 2023, the country documented 19 anthrax outbreaks across various regions, predominantly within the cattle corridor that spans the north, east, and west(7–10). Surveillance data from this period reported 186 human and 721 livestock deaths attributed to anthrax (11).

On December 28, 2023, a 10-year-old child from Kakworobu Village, a rural community in Amudat District, sought medical attention at a local health center with symptoms consistent with anthrax, including an eschar, body weakness, and itching on the shoulders. The health center, , was the first to identify suspected cases of anthrax,, signaling the onset of a potential outbreak. Sporadic cases continued to appear into early March 2024, prompting health authorities to alert district officials on March 5, 2024. On

March 25, 2024, laboratory tests confirmed the presence of anthrax in human samples. This marked the first known anthrax outbreak in the district, highlighting the urgent need for a public health response. We investigated to assess the outbreak's magnitude, identify associated risk factors, and recommend control and prevention measures.

Methods

Outbreak area

Amudat District is located in north-eastern Uganda, within the Karamoja sub-region. shares borders with Kenya to the east, Moroto District to the north, Nakapiripirit to the west, and Kween and Bukwo District to the south. The district has a population of approximately 157,800 according to Uganda Bureau of Statistics (UBOS) and people are predominantly nomadic pastoralists. The district has 10 sub-countries including Amudat Town Council, Loroo, Karita Town Council, Abiliyep, Katabok, Karita, Kangorok, Lokales, Losidok, and Achorichori (**Fig 1**).

Fig 1: Location of Amudat District in Uganda

Case definition and finding

We defined a suspected case of cutaneous anthrax as acute onset of skin lesions (vesicle or eschar) plus ≥2 of skin itching, reddening, and swelling, or regional lymphadenopathy that occurred in a resident of Amudat District from December 2023–June 2024.

A confirmed case was a suspected case testing PCR-positive for *Bacillus anthracis.*

To identify cases, we reviewed health facility records at health facilities serving the affected community and conducted an active community case searches in collaboration with the community health workers.

Descriptive epidemiology

We performed descriptive epidemiology on the line-listed case-patients. Case-patients were described by time, place, person characteristics. We constructed an epidemic curve to depict the distribution of cases over time. Attack rates were calculated using Uganda Bureau of Statistics (UBOS) population estimates, stratified by age group, sex, and sub-county. Choropleth maps were developed to illustrate the geographic distribution of cases across sub-counties.

Laboratory investigations

Samples from human skin lesions, cattle carcasses and soil were collected and transferred to the testing laboratory using the hub system (a centralized network designed to efficiently transport medical specimens from health facilities to laboratories for analysis). The human samples were triple-packaged and transported to the Uganda Virus Research Institute (UVRI) laboratory in Arua District. The animal and environmental samples were transported to the National Animal Disease Diagnostics and Epidemiology Centre (NADDEC) laboratory in Entebbe, Uganda. Confirmation of anthrax was based on PCR identification of *Bacillus anthracis*.

Environmental investigations

We inspected grazing sites and areas where cattle had died suddenly, focusing on carcass disposal practices to identify potential contamination sources. We also assessed the livestock vaccination status within the affected sub-counties.

Hypothesis generation

To generate hypotheses, we conducted interviews with 40 case-patients to identify various exposures associated with contracting anthrax. These exposures included slaughtering cattle that had died suddenly, sleeping on the hides of these cattle, and digging up buried animal remains during farming.

Case-control study

We carried out an unmatched case-control study with a 1:3 ratio, involving 40 cases and 120 controls to identify risk factors for anthrax infection. All confirmed and suspected cases from December 2023 to June 2024 were enrolled. The rest of the case-patients were not included due to the nomadic nature of the Pokot population, which made it challenging to locate and follow up on some case-patients, especially those who had migrated or were unreachable during the study period. We randomly selected controls from the nearest household of cases and were screened for the clinical signs and symptoms before considering them eligible. Using structured questionnaires, we collected demographic characteristics (age, sex, and sub-county) and potential exposures (slaughtering cattle that had died suddenly, skinning infected cattle, carrying the meat of cattle that died suddenly, sleeping on the hides of cattle that died suddenly, dug out animal remains).

Data were analyzed using Epi Info software. Crude odds ratios (OR) were computed at the bivariate analysis. Factors found significant at this level were included in a multivariable logistic regression model to calculate adjusted odds ratios (aOR), with significance set at $p<0.05$. A common reference group analysis was also conducted to evaluate risk associated with multiple exposure combinations(12).

Ethical considerations

This outbreak investigation was in response to a public health emergency and was therefore determined to be non-research. The Ministry of Health (MoH) gave permission to investigate this outbreak. In agreement with the International Guidelines for Ethical Review of Epidemiological Studies by the Council for International Organizations of Medical Sciences (1991) and the Office of the Associate Director for Science, US CDC/Uganda, it was determined that this activity was not human subject research and that its primary intent was public health practice or disease control activity (specifically, epidemic or endemic disease control activity). This activity was reviewed by the US CDC and was conducted consistent with applicable federal law and CDC policy. §§See, e.g., 45 C.F.R. part 46, 21 C.F.R. part 56; 42 U.S.C. §241(d); 5 U.S.C. §552a; 44 U.S.C. §3501 et seq. All experimental protocols were approved by the US CDC human subjects review board (The National Institute for Occupational Safety and Health Institutional Review Board) and the Uganda MoH and were performed in accordance with the Declaration of Helsinki. Permission to conduct the outbreak response was also granted by Amudat District Local Government. Prior to data collection, informed consent was obtained from all the participants who were aged 18 years or older (legal age in Uganda). For those below 18 years, consent was sought from their parents/guardians and assent was also obtained from them to participate in the study.

Results

Descriptive epidemiology

We identified 102 case-patients, 40 of whom were confirmed, with no fatalities. The overall attack rate was 167 per 100,000 population, with all cases presenting in the cutaneous form.

The mean age of the 40 confirmed cases was 24 years (range: 7 months–70 years), with the 5–20 years age group being the most affected (attack rate: 206/100,000), and the under 5 years group the least affected (attack rate: 132/100,000). Males had a higher attack rate (201/100,000) compared to females (132/100,000). Geographically, Losidok Sub-county recorded the highest attack rate (350/100,000), whereas Abiliyep Sub-county had the lowest (8 / 100,000) (**Table 1).**

Table 1: Attack rates by age, sex, sub-county among case-patients during a cutaneous anthrax outbreak, Amudat District, December 2023–June 2024

Variables	Cases	Population	AR/100,000
**Age**			
<5 years	13	9,866	132
5-20 years	47	22,776	206
> 20 years	42	28,258	149
**Sex**			
Male	63	31,400	201
Female	39	29,500	132
**Sub-county**			
Losidok	49	14,000	350
Karita	41	14,100	291
Lokales	10	16,800	60
Karita TC	1	4,100	24
Abiliyep	1	11,900	8
**Total**	**102**	**60,900**	**167**

AR: Attack rate**; TC:** Town Council

Among 40 case-patients, skin itching (75%), skin swelling (73%) and eschar (70%) were the most common signs and symptoms of illness. (**Fig 2)**.

Fig 2: Distribution of clinical symptoms of anthrax case-patients during an anthrax outbreak, Amudat District, December 2023–June 2024 (n=40)

On December 22, 2023, cow A died in neighboring Kween District. It was then slaughtered, and its meat transported to Amudat; two days later, the first suspected human case emerged with signs and symptoms suggestive of cutaneous anthrax and visited Karita HCIV in Karita Sub-county on December 28, 2023. On January 20, 2024, cow B died in Karita Sub-county, and shortly thereafter, additional human cases emerged. Towards the end of February 2024, over 100 cattle died, this was the time cattle were returning from Kween District and it was followed by marked increase in human cases. On March 3, 2024, the index case was identified, prompting an alert to the district, on March 4, 2024, the Moroto Regional Emergency Operations Centre (REOC) was notified. On March 15, 2024, a joint team from the district and REOC verified the alerts at Karita HC4. It took 66 days to detect the outbreak, one day to be notified and 12 days for the district to initiate response.

The cases were initially confined to Karita Sub-county but later spread to Losidok and Lokales Sub-counties. Cattle movement in search of pasture, particularly during periods of drought or limited grazing areas, likely contributed to the spread of anthrax as they roamed into areas with infected animals or contaminated grazing grounds.

A noticeable surge that occurred in March 2024, prompted the district to respond on March 15,2024. The carcasses of infected animals were safely disposed of to prevent

further spread, and awareness campaigns were conducted in the affected sub-counties to help reduce transmission (**Fig 3**).

Fig 3: Distribution of cutaneous anthrax cases across sub-counties, Amudat District, December 2023–June 2024

Laboratory findings

Among 16 human skin lesion swabs collected, seven (44%) tested positive for B. anthracis by PCR at UVRI. The remaining 9 samples were negative. All six samples from the cattle carcasses and two soil samples tested negative at NADDEC.

Environmental assessment findings

In Losidok Sub-county, we observed scattered cattle bones, suggesting prior improper disposal of animal carcasses.

We also came across discarded hides, which appeared to have been used as bedding and were found near residential areas in Karita. Additionally, none of the respondents reported vaccinating their livestock against anthrax before the onset of the outbreak.

Hypothesis generation findings

Of the 40 participants interviewed during hypothesis generation, 33(83%) reported slaughtering cattle that had died suddenly. Additionally, 33% respondents indicated they were involvement in digging up animal remains (bones) during cultivation and 30% admitted to sleeping on the hides. We therefore hypothesized that sleeping on cattle hides and slaughtering the animals that died suddenly may have been associated with the anthrax outbreak in Amudat District (**Table 2).**

Table 2: Hypothesis generation among case-patients during a cutaneous anthrax outbreak, Amudat District, December 2023–June 2024 (n-40)

Exposure	n	(%)
Slaughter of cattle that had died suddenly	33	(83%)
Dug out the animal remains during cultivation	13	(33%)
Slept on hides of dead cattle that died suddenly	12	(30%)
Participated in carrying of meat	6	(15%)

Case control study findings

Residents who slept on the hide of the cattle that had died suddenly were 11 times more likely to contract cutaneous anthrax compared to those who did not (aOR = 11, 95% CI: 2.6-47). Furthermore, individuals who slaughtered the cattle that had died suddenly were 7.6 times more likely to develop cutaneous anthrax compared to those who did not (aOR = 7.6, 95% CI: 2.1-28) **(Table 3).**

Table 3: Exposure factors associated with an anthrax outbreak, Amudat District, Uganda, December 2023–June 2024

	Number of participants			
**Exposures**	**Cases (%)**	**Control (%)**	**cOR (95%, CI**	**aOR (95%, CI**
**Age**				
<5 years	6(30)	3(15)	Ref	
5-20 years	14(70)	17(85)	2.4(0.4–17)	3.7(1.2–11)
>20 years	20(77)	100(97)	10(1.9–65)	20.1(3.3–121)
**Sex**				
Male	24(60)	55(46)	1.8(0.9–3.7)	
Female	16(40)	66(54)	Ref	
**Contact with the hide**				
Yes	24(60)	32(27)	4.1(1.9–8.7)	
No	16(40)	88(73)	Ref	
**Dug-out animal remains**				
Yes	13(32.5)	21(17.5)	2.3(1.0–5.1)	
No	27(67.5)	99(82.5)	Ref	
**Sleeping on the**				

**hide of a dead cow**				
Yes	12(30)	3(2.5)	17 (4.0–96)	11(2.6–47)
No	28(70)	117(97.5)	Ref	
**Skinning of the cattle**				
Yes	13(32.5)	10(14.4)	5.3(2.1–13)	
No	27(67.5)	110(85.6)	Ref	
**Participated in slaughtering**				
Yes	33(82.5)	41(34.2)	9.1(3.7–22)	7.6 (2.1–28)
No	7(17.5)	79(65.8)	Ref	

Ref: reference; cOR: crude odds ratios; aOR: adjusted odds ratios; CI: confidence interval

Compared to the unexposed group (those with no contact with the dead cattle), individuals who engaged in more exposure activities had higher odds of contracting anthrax. Those who only slaughtered had an odds ratio (OR) of 6.9 (95% CI: 2.6–18). The odds were higher for those who slaughtered and carried cattle parts (OR=11, 95% CI: 1.2–96), slaughtered and skinned (OR=14, 95% CI: 3.5–56), and for those who performed all three activities—slaughtering, carrying cattle parts, and skinning (OR=17, 95% CI: 1.6–219) **(Table 4).**

Table 4: Common reference analysis of risk factors associated with cutaneous anthrax, Amudat, December 2023–June 2024

Carried	Skinned	Slaughtered	OR	95%CI
-	-	-	Ref	
-	-	+	6.9	2.6-18
+	-	+	11	1.2-96

-	+	+	14	3.5-56
+	+	+	17	1.6-219

OR: odds ratio; CI: confidence interval

Discussion

This outbreak represents the first documented case of anthrax in Amudat District. Our investigation identified key risk factors, including direct exposure to animal hides and involvement in slaughtering activities, with a synergistic effect of these exposures amplifying the cumulative risk. Additionally, there was a lack of prior vaccination among livestock in the district. The outbreak affected five sub-counties and no death was reported. While the outbreak response faced significant delays in detection and response, the one-day notification requirement for the 7-1-7 metrics was met.

Our findings suggest a multi-source outbreak of cutaneous anthrax, linked to direct contact with or handling of deceased cattle, with sleeping on cattle hides emerging as a key risk factor. In the Pokot community, young children, particularly those involved in cattle herding, commonly use hides as bedding, which increases the likelihood of exposure to *Bacillus anthracis* spores. This practice aligns with previous studies which have highlighted the role of cattle hides in anthrax transmission among vulnerable populations (13,14).

Notably, this is the first documented outbreak in Uganda where sleeping on hides from suddenly deceased cattle has been identified as a significant risk factor.

These findings underscore the need for greater awareness regarding this practice and the implementation of safer alternatives. Involving community and cultural leaders in

promoting preventive measures will be key to ensuring that these messages are effectively communicated. Slaughtering of deceased anthrax-infected animals was also a key factor in the transmission of anthrax to humans. These findings are consistent with previous studies that have identified exposure to infected animals or animal products as a significant risk factor for human anthrax (11,15–17). Given the risk of recurrent outbreaks in Uganda, ongoing community education on safe carcass disposal is essential to prevent future outbreaks.

The findings from the 7-1-7 metrics showed a two-month delay in detecting and responding to the anthrax outbreak in Amudat District. This delay mirrors challenges faced globally in managing public health threats (18,19). To improve global health security, it is essential to strengthen early detection systems, enhance surveillance, train local health workers, and ensure better communication and coordination among stakeholders for faster, more effective responses to future outbreaks.

Our findings have important public health implications for preventing future anthrax outbreaks in the region. First, the identification of key risk factors, such as sleeping on cattle hides and handling meat from deceased cattle, highlights the need for targeted interventions in nomadic pastoral communities to reduce exposure. Health education campaigns should focus on raising awareness about the dangers of these practices, particularly the use of cattle hides as bedding and handling meat from animals that have suddenly died. Second, to mitigate the risk of transmission from livestock to humans, improving animal surveillance and vaccination programs is crucial. Routine vaccination of livestock should be prioritized, as our investigation revealed that none of the animals

in the affected district were vaccinated prior to the outbreak. Finally, engaging community and cultural leaders is essential in promoting safer practices and fostering behavioral change, especially in high-risk nomadic communities.

One key limitation of this study is the likely underreporting of gastrointestinal (GI) anthrax cases, as symptoms may mimic other common GI conditions, leading to misdiagnosis. This, combined with the unfamiliarity of local healthcare providers with anthrax, given its novelty in the district likely delayed recognition and reporting, resulting in an underestimation of the outbreak's true magnitude.

Conclusion

This outbreak of cutaneous anthrax was primarily linked to handling of animal hides and the slaughter of cattle that had died suddenly, with detection and response hindered by delayed recognition. To mitigate future outbreaks, we recommend enhancing community awareness about anthrax transmission, ensuring timely vaccination of livestock, and promoting safe practices for carcass handling and disposal. Strengthening integrated surveillance systems across human and animal health sectors, in accordance with the One Health framework, will be vital for improving early detection and response to similar zoonotic threats.

List of abbreviations

AR: attack rate; aOR: adjusted odds ratio; CDC: Centers for Disease Control and Prevention; CI: confidence interval; cOR: Crude Odds Ratio; VHTs: Village Health Teams; UBOS: Uganda Bureau of Statistics; UVRI: Uganda Virus Research Institute; NADDEC: National Animal Disease Diagnostics and Epidemiology Centre; WHO: World Health Organization; MoH: Ministry of Health

Declarations

Ethics and consent to participate

This outbreak investigation was in response to a public health emergency and was therefore determined to be non-research and therefore clinical trial number not applicable. The Ministry of Health (MoH) gave permission to investigate this outbreak. In agreement with the International Guidelines for Ethical Review of Epidemiological Studies by the Council for International Organizations of Medical Sciences (1991) and the Office of the Associate Director for Science, US CDC/Uganda, it was determined that this activity was not human subject research and that its primary intent was public health practice or disease control activity (specifically, epidemic or endemic disease control activity). This activity was reviewed by the US CDC and was conducted consistent with applicable federal law and CDC policy. §§See, e.g., 45 C.F.R. part 46, 21 C.F.R. part 56; 42 U.S.C. §241(d); 5 U.S.C. §552a; 44 U.S.C. §3501 et seq. All experimental protocols were approved by the US CDC human subjects review board (The National Institute for Occupational Safety and Health Institutional Review Board) and the Uganda Ministry of Health and were performed in accordance with the Declaration of Helsinki. Prior to data collection, informed consent was obtained from all the participants who were above 18 years (legal age in Uganda). For those below 18 years, consent was sought from their parents/guardians and assent was also obtained from them to participate in the study.

Availability of data and materials

The datasets upon which our findings are based belong to the Uganda Public Health Fellowship Program. For confidentiality reasons, the datasets are not publicly available. The datasets can be availed upon reasonable request from the corresponding author

with permission from the Uganda Public Health Fellowship Program. Request can be directed at: pkwizera@uniph.go.ug

Acknowledgments

We extend our appreciation to the District Health Team of Amudat, particularly the District Surveillance Focal Person and the surveillance team for their overall coordination and leadership during the investigation in the district. We also thank Karita Health Centre IV for their coordination with the Village Health Teams, and acknowledge the Village Health Teams for their active participation in the case search within the community. Lastly, we recognize the Uganda Public Health Fellowship Program for providing technical oversight, coordination, and throughout the investigation.

Author contribution

PK, conceptualized the study idea, collected data, analyzed it and wrote the manuscript HK, EN, JM, RM conceptualized the study idea, collected data and reviewed the manuscript. SG, RM, BK, and ARA supported in editing, and reviewing of the manuscript. All authors read and approved the final manuscript.

References

1. Alam M, Kamal M, Rahman M, Kabir A, Islam M, Hassan J. Review of anthrax: A disease of farm animals. J Adv Vet Anim Res. 2022;9(2):323.

2. Zasada A. Injectional anthrax in human: A new face of the old disease. Adv Clin Exp Med. 2018 Apr 30;27(4):553–8.

3. Nayak P, Sodha SV, Laserson KF, Padhi AK, Swain BK, Hossain SS, et al. A cutaneous Anthrax outbreak in Koraput District of Odisha-India 2015. BMC Public Health. 2019 May;19(S3):470.

4. Kasradze A, Echeverria D, Zakhashvili K, Bautista C, Heyer N, Imnadze P, et al. Rates and risk factors for human cutaneous anthrax in the country of Georgia: National surveillance data, 2008–2015. Duesbery NS, editor. PLoS ONE. 2018 Feb 7;13(2):e0192031.

5. Badri R, Uwishema O, Wellington J, Thambi VD, Pradhan AU, Adanur I, et al. Anthrax outbreak amidst the COVID-19 pandemic in Africa: Challenges and possible solutions. Annals of Medicine & Surgery [Internet]. 2022 Sep [cited 2024 Jul 1];81. Available from: https://journals.lww.com/10.1016/j.amsu.2022.104418

6. Sekamatte M, Krishnasamy V, Bulage L, Kihembo C, Nantima N, Monje F, et al. Multisectoral prioritization of zoonotic diseases in Uganda, 2017: A One Health perspective. Kuhn JH, editor. PLoS ONE. 2018 May 1;13(5):e0196799.

7. Nuwamanya Y, Kizza D, Kiyimba A, Ssemanda I, Aanyu D, Monje F, et al. Anthrax outbreaks in western Uganda: The role of illegal meat dealers in spreading the infection, August 2022–April 2023. 2024;9(1).

8. Migisha R, Mbatidde I, Agaba DC, Turyakira E, Tumwine G, Byaruhanga A, et al. Risk factors for human anthrax outbreak in Kiruhura District, Southwestern Uganda: a population-based case control study. 2021 [cited 2024 Jun 29]; Available from: https://nru.uncst.go.ug/handle/123456789/6290

9. Kabami Z, Agaba B, Naiga HN, Simbwa BN, Zavuga R, Kizito SN, et al. Emergence of anthrax in Bududa District, Eastern Uganda, February - May 2022: implications for prevention and control. PAMJ-OH [Internet]. 2024 [cited 2025 Mar 23];14. Available from: https://www.one-health.panafrican-med-journal.com/content/article/14/15/full

10. Nakanwagi M, Ario AR, Kwagonza L, Aceng FL, Mwesigye J, Bulage L, et al. Outbreak of gastrointestinal anthrax following eating beef of suspicious origin: Isingiro District, Uganda, 2017. PLoS Negl Trop Dis. 2020 Feb;14(2):e0008026.

11. Musewa A, Mirembe BB, Monje F, Birungi D, Nanziri C, Aceng FL, et al. Outbreak of cutaneous anthrax associated with handling meat of dead cows in Southwestern Uganda, May 2018. Trop Med Health. 2022 Aug 6;50(1):52.

12. Fontaine RE, He Y, Zhu BP. Untangling the effects of multiple exposures with a common reference group in an epidemiologic study: A practical revisit. PLoS One. 2023 Dec 15;18(12):e0295915.

13. Mwakapeje ER, Høgset S, Softic A, Mghamba J, Nonga HE, Mdegela RH, et al. Risk factors for human cutaneous anthrax outbreaks in the hotspot districts of Northern Tanzania: an unmatched case–control study. R Soc open sci. 2018 Sep;5(9):180479.

14. Munang'andu HM, Banda F, Chikampa W, Mutoloki S, Syakalima M, Munyeme M. Risk analysis of an anthrax outbreak in cattle and humans of Sesheke district of Western Zambia. Acta Tropica. 2012 Nov;124(2):162–5.

15. Aceng FL, Ario AR, Alitubeera PH, Neckyon MM, Kadobera D, Sekamatte M, et al. Cutaneous anthrax associated with handling carcasses of animals that died suddenly of unknown cause: Arua District, Uganda, January 2015–August 2017. Beechler BR, editor. PLoS Negl Trop Dis. 2021 Aug 23;15(8):e0009645.

16. Ntono V, Eurien D, Bulage L, Kadobera D, Harris J, Ario AR. Cutaneous anthrax outbreak associated with handling dead animals, Rhino Camp sub-county: Arua District, Uganda, January–May 2018. One Health Outlook. 2021 Dec;3(1):8.

17. Kisaakye E, Ario AR, Bainomugisha K, Cossaboom CM, Lowe D, Bulage L, et al. Outbreak of Anthrax Associated with Handling and Eating Meat from a Cow, Uganda, 2018. Emerg Infect Dis. 2020 Dec;26(12):2799–806.

18. Bochner AF, Makumbi I, Aderinola O, Abayneh A, Jetoh R, Yemanaberhan RL, et al. Implementation of the 7-1-7 target for detection, notification, and response to public health threats in five countries: a retrospective, observational study. The Lancet Global Health. 2023 Jun 1;11(6):e871–9.

19. Frieden TR, Lee CT, Bochner AF, Buissonnière M, McClelland A. 7-1-7: an organising principle, target, and accountability metric to make the world safer from pandemics. Lancet. 2021;398(10300):638–40.

Manuscript Click here to access/download;Figure;Figures.docx

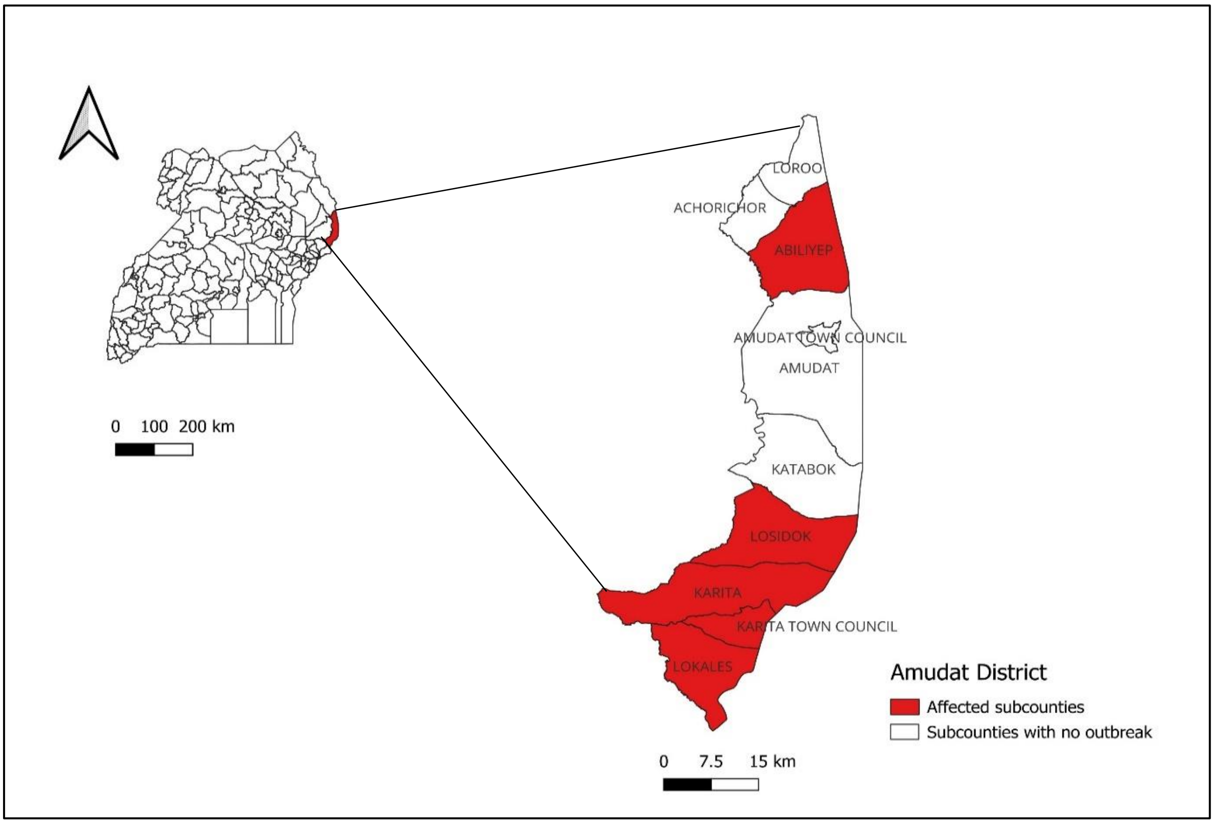

Fig 1: Location of Amudat District in Uganda

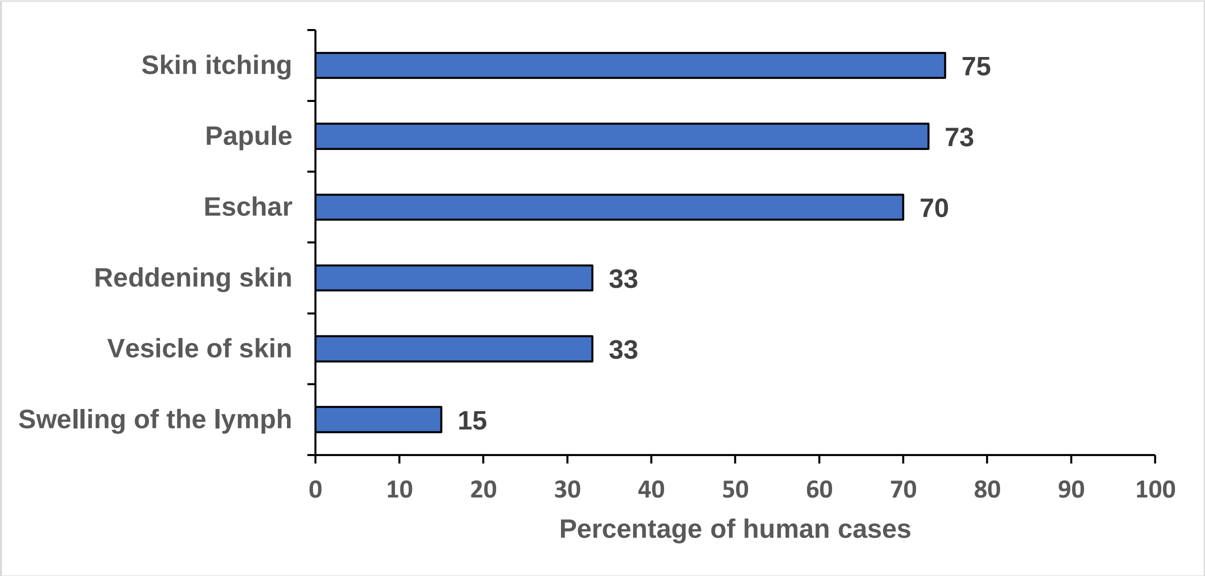

Fig 2*: *Distribution of clinical symptoms of anthrax case-patients during an anthrax outbreak, Amudat District, December 2023–June 2024 (n=40)

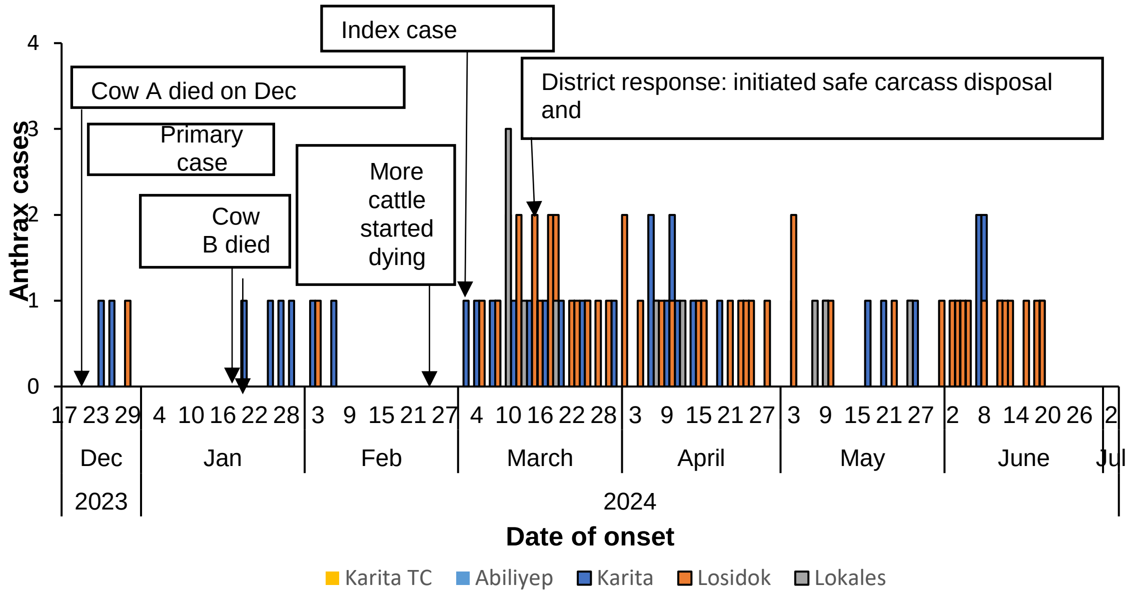

***Fig 3*: Distribution of cutaneous anthrax cases across sub-counties, Amudat District, December 2023–June 2024**
