# Supplementary material for: Cutaneous anthrax outbreak associated with use of cattle hides and handling carcasses, Amudat District, Uganda, 2023–2024
Source: PLoS One. 2025 Nov 17;20(11):e0336769. doi: 10.1371/journal.pone.0336769 (PMC12622773; doi:10.1371/journal.pone.0336769)
Supplement: Attachment — Submitted filename: Response to reviewers about data availability.docx [file pone.0336769.s005.docx]

September 4, 2025

Editor, Plos One

Dear Sir/Madam

**RE: Cutaneous anthrax outbreak linked to using cattle hides as bedding and handling carcasses, Amudat District, Uganda, December 2023–June 2024**

We sincerely thank the reviewers and the Editor for their thorough review and constructive remarks. We have carefully considered and addressed all comments in the revised manuscript

**Comment**

In this instance it seems there may be acceptable restrictions in place that prevent the public sharing of your minimal data. However, in line with our goal of ensuring long-term data availability to all interested researchers, PLOS’ Data Policy states that authors cannot be the sole named individuals responsible for ensuring data access (<http://journals.plos.org/plosone/s/data-availability#loc-acceptable-data-sharing-methods>).
Data requests to a non-author institutional point of contact, such as a data access or ethics committee, helps guarantee long term stability and availability of data. Providing interested researchers with a durable point of contact ensures data will be accessible even if an author changes email addresses, institutions, or becomes unavailable to answer requests.
Before we proceed with your manuscript, please also provide non-author contact information (phone/email/hyperlink) for a data access committee, ethics committee, or other institutional body to which data requests may be sent. If no institutional body is available to respond to requests for your minimal data, please consider if there any institutional representatives who did not collaborate in the study, and are not listed as authors on the manuscript, who would be able to hold the data and respond to external requests for data access? If so, please provide their contact information (i.e., email address). Please also provide details on how you will ensure persistent or long-term data storage and availability.

**Response**

We thank the Editors and Reviewers for this important guidance regarding long-term data access and availability. We fully acknowledge and respect PLOS’ Data Policy, and we have taken steps to ensure compliance.

For this study, the dataset is stored securely with the **Uganda Public Health Fellowship Program (UPHFP)** under the **Uganda National Institute of Public Health (UNIPH).** Data requests can be directed to institutional information department headed by Khoba Barbara on email: it@uniph.go.ug

This arrangement ensures that data will remain available beyond the tenure of individual authors, and requests will be handled in a consistent and transparent manner. The data are stored on secure institutional servers with guaranteed long-term preservation in accordance with Uganda Ministry of Health and UNIPH data governance policies.

We hope this satisfies the journal’s requirements and are happy to provide any additional details needed.

We thank you for your time in invested in reviewing our manuscript and guidance you have continued to provide.
