# Supplementary material for: Cutaneous anthrax outbreak associated with use of cattle hides and handling carcasses, Amudat District, Uganda, 2023–2024
Source: PLoS One. 2025 Nov 17;20(11):e0336769. doi: 10.1371/journal.pone.0336769 (PMC12622773; doi:10.1371/journal.pone.0336769)
Supplement: Attachment — Submitted filename: Response to reviewers v2.docx [file pone.0336769.s006.docx]

October 28, 2025

Prof. Chisoni Mumba
Academic Editor

PLOS ONE

**RE:** **Cutaneous anthrax outbreak associated with use of cattle hides and handling carcasses, Amudat District, Uganda, 2023–2024**

Dear Prof. Chisoni Mumba

We sincerely thank the reviewers and the Editor for their thorough review and constructive remarks. We have carefully considered and addressed all comments in the revised manuscript.

We have carefully considered and addressed all comments in the revised manuscript. Below, we provide a detailed, point-by-point response indicating how each comment was addressed in the revised version.

We acknowledge the efforts of the reviewers and the Editor. For any further clarifications and information, please don’t hesitate to contact us.

Sincerely,

Patrick Kwizera (Corresponding Author)

**RESPONSE TO REVIEWERS’ COMMENTS**

**Editors comments:**

Your title is not concise, please revise it. some suggestions, "Cutaneous Anthrax Outbreak Associated with Use of Cattle Hides and Carcass Handling in Amudat District, Uganda (2023-2024".

The title and discussion still over-interpret the role of sleeping on cattle hides as a proven route of transmission, while laboratory evidence (soil = negative; hides = not tested or negative) does not confirm this pathway. The association rests solely on self-reported exposure and a small sample with wide CIs (OR = 11, 95 % CI 2.6–47). The claim of being “the first outbreak linked to use of hides as bedding in Uganda” should therefore be framed cautiously as “epidemiologically associated” rather than “linked” or “caused.” Given that the evidence is epidemiological (associative), without laboratory confirmation of the hides or hides testing positive for Bacillus anthracis, the title should reflect association, not causation.

reported outbreak.
**Response**

We appreciate this important clarification. The title has been revised to: Cutaneous Anthrax Outbreak Associated with Use of Cattle Hides and Carcass Handling in Amudat District, Uganda (2023–2024) to align with your suggestion and to use epidemiologically neutral language that reflects association rather than causation throughout the manuscript

We thank you once again for your valued time you invested in reviewing our manuscript.
